# The DSM validation method and its *decision points*: value judgments, metaphysical presuppositions, and validators

**DOI:** 10.1007/s40656-026-00728-3

**Published:** 2026-04-15

**Authors:** Antonio Rodríguez Puente

**Affiliations:** Department of Philosophy, Metropolitan Autonomous University, Mexico City, Mexico

**Keywords:** Validation, Decision points, Non-empirical factors, Value judgments, Metaphysical assumptions, General models

## Abstract

This paper offers an analysis of the DSM validation method, focusing on the role that non-empirical factors play in resolving forms of indeterminacy that cannot be settled by validator evidence alone. It begins by reconstructing the historical development and institutionalization of the DSM validation method, showing that although it emerged as an attempt to ground the classification of mental disorders in empirical evidence rather than in a priori preconceptions or contextual influences, it nonetheless came to rely on extra-empirical considerations at several crucial stages and was, in its very design, shaped by them.The paper addresses this tension by drawing on the concept of a *decision point* developed in the literature on science and values. By identifying a series of decision points that arise both in the application of the validation method to particular diagnostic categories and in the process of designing and developing the method itself, it argues that the incorporation of value judgments and metaphysical presuppositions constitutes a necessary and epistemically pertinent element that enabled the validation method to take shape and, when applied to specific categories, allows the validation process to move beyond its initial stages and reach a determinate conclusion.

## Introduction

In psychiatric nosology, *validation* refers to the process through which diagnostic categories of mental disorders are formed, assessed, and, in some cases, incorporated into official classificatory systems. In both the philosophical and psychiatric literature, validation is often discussed alongside related but distinct concepts, including validity, reliability, and utility. *Validity* is commonly understood as the property of a diagnostic category to correspond to a real nosological entity; however, it remains a contested and metaphysically laden notion that lacks a widely accepted definition (Kendell & Jablensky, 2003; Solomon, 2022). *Validation*, by contrast, concerns the evidential assessment of diagnostic categories by means of specific *validators*, or sources of empirical evidence (Robins & Guze, 1970; Kendler, 1980; APA DSM Team, 2021). *Reliability* refers to the degree of agreement among clinicians applying the same diagnostic criteria and does not, by itself, guarantee correspondence with a real disorder (Murphy, 2014). *Utility*, finally, denotes the practical value of a diagnosis for clinical, research, educational, or administrative purposes and should not be conflated with either empirical validation or ontological validity (Kendell & Jablensky, 2003). This paper focuses on validation as a method, while keeping these distinctions in view.

Over the past decades, several conceptions of psychiatric validation have been proposed. Among these, the framework developed by Robins and Guze (1970) and later refined by Kendler (1980) has gained sufficient traction within the mental health community to become institutionalized in the official guidelines of the *Diagnostic and Statistical Manual of Mental Disorders* (DSM), currently the most widely used and internationally influential system for psychiatric classification (APA, 1980, 1994, 2013). This framework characterizes validation as a process in which hypotheses about diagnostic categories are evaluated on the basis of evidence derived from multiple types of empirical studies, or *validators*, and it now defines the methodological standards governing proposals for diagnostic change within the DSM (APA DSM Team, 2021).

The incorporation of this version of the validation method into DSM institutional practices was neither straightforward nor immediate, but rather a gradual process shaped by historical, theoretical, and institutional factors. Its original development responded to the perception that psychiatric classification had long relied on a priori assumptions, clinical traditions, and contextual influences rather than on systematic assessments of empirical evidence. Against this background, the framework proposed by Robins and Guze (1970) and refined by Kendler (1980) was intended to anchor psychiatric classification more firmly in empirical findings.

Yet, as this method was developed and applied, it became increasingly clear that the validation of psychiatric categories could not proceed on evidential grounds alone. Decisions concerning the prioritization of different sources of evidence, the resolution of conflicts among validators, and the setting of evidential thresholds required the incorporation of factors not themselves determined by empirical data. This tension is further compounded by the fact that key decisions in the design of the DSM validation method—including those concerning the *general models* of mental disorder that structure diagnostic constructs and guide evidential expectations—were also influenced by non-empirical considerations.

In this paper, I address this tension by analyzing the stages of the DSM validation process at which non-empirical factors enter into play and by clarifying the roles they serve. Drawing on the notion of *decision point*, as defined by Brown (2020), I examine how both *value judgments* and *metaphysical presuppositions* shape psychiatric validation. On this basis, I argue that the incorporation of these types of non-empirical factors into the DSM validation method can be understood as *epistemically pertinent* and, under certain conditions, as a necessary condition for advancing beyond the initial stages of validation and reaching a conclusion.

## The development of the DSM validation method

This section examines several key moments in the historical development of the DSM validation method, with particular attention to how the aims of the validation process were understood at different stages. It also considers the prevailing attitudes toward the incorporation of non-empirical factors, as well as the specific factors that guided decision-making during these periods.

### Robins and Guze’s five phases of validation

The first systematic attempt to develop a method for validating psychiatric categories can be traced to the seminal paper “Establishment of Diagnostic Validity in Psychiatric Illness: Its Application to Schizophrenia,” by Eli Robins and Samuel Guze (1970). In this work, they outlined five “phases” through which a diagnostic category is validated: *(1) clinical description*,* (2) laboratory studies*,* (3) delimitation from other disorders*,* (4) follow-up studies*,* and (5) family studies*. Of these five phases, only *clinical description* and *delimitation from other disorders* properly function as stages of a research method. The remaining phases are better understood as referring to types of studies that provide evidence for the validity of diagnostic categories, a distinction that to some extent anticipates the later notion of a *validator*.

A further feature of Robins and Guze’s account is that these phases are conceived as standing in a feedback relationship, such that findings obtained in one phase may lead to revisions in others: “The entire process is therefore one of continuing self-rectification and increasing refinement leading to more homogeneous diagnostic grouping” (Robins & Guze, 1970, p. 984). Accordingly, several authors have emphasized that the phases form a *continuous* and *iterative* process rather than a strictly linear sequence (Schaffer, 2012; Surís et al., 2016).

This iterative conception is connected to an aspect of Robins and Guze’s framework that would later recede from prominence, namely, that validation is not restricted to testing already well-defined diagnostic categories against evidence from specific study types. Instead, the process also encompasses the earlier activity of *construct formation*, captured by the *clinical description phase*, as well as the subsequent refinement of categories through *delimitation from other disorders*. On this view, a lack of supporting evidence does not terminate the validation process but rather guides the reformulation of the category, thereby reopening the phase of clinical description.

With respect to the outcome of the validation process, Robins and Guze (1970, p. 983) characterize it as the establishment of the *diagnostic validity* of psychiatric categories, although they do not offer an explicit account of this notion. Nonetheless, their understanding of diagnostic validity can be inferred from the kind of categories their method is expected to yield. In their view, valid categories take the form of progressively more *homogeneous groups*, where homogeneity is defined in terms of clinical presentation, outcome, and underlying etiology. The production of such homogeneous groups was taken not only to increase internal consistency but also to enable more precise etiological and follow-up studies (Robins & Guze, 1970, p. 984).

The validation method proposed by Robins and Guze is best understood as part of a broader program initiated by a group of researchers at the University of Washington in St. Louis, aimed at legitimizing *psychiatric nosology* (i.e., the branch of psychiatry dealing with the classification of mental disorders) as a scientific enterprise (Kendler et al., 2010; Hyman, 2010; Kendler, 2017). This program was motivated by the diagnosis that psychiatric classification had long relied on non-scientific practices, a point explicitly articulated by Robins and Guze:

One of the reasons that diagnostic classification has fallen into disrepute among some psychiatrists is that diagnostic schemes have been largely based upon *a priori principles* rather than upon *systematic studies*… We have found that the approach described here facilitates the development of a valid classification in psychiatry. (Robins & Guze, 1970, p. 983, *emphasis added*)

Accordingly, the Washington University group criticized the widespread practice of assessing classificatory decisions by their conformity with *a priori conceptions* rather than by the amount and quality of the supporting evidence. Robins and Guze thus conceived their method as a corrective measure, insofar as it aimed to ground psychiatric categories in a cumulative body of empirical findings.

Over the following decade, the five phases proposed by Robins and Guze were employed, albeit somewhat loosely, as the methodological framework for the validation of the classificatory system developed by the Washington University group. In a highly influential publication, this group introduced the *Feighner criteria* (Feighner et al., 1972), a set of operational diagnostic criteria intended to improve the reliability of psychiatric diagnoses in both clinical and research settings. These criteria subsequently informed the development of the *Research Diagnostic Criteria* (Spitzer et al., 1978) and later the *DSM-III* (APA, 1980), which considerably expanded the number of recognized diagnostic categories. Despite its influence, the Robins and Guze method was neither formally institutionalized nor systematically applied in the development of the *DSM-III* or its subsequent revisions (Kendler et al., 2010; Solomon, 2022).

### Kendler’s eight validators

Ten years after the publication of Robins and Guze’s article, Kenneth Kendler presented what he described as an “adaptation and enlargement” of their original validation method (1980, p. 700). The most salient innovation in Kendler’s proposal was the substitution of the notion of *phase* with that of *validator*, reflecting a heightened emphasis on the different kinds of evidence that could be brought to bear in the validation of diagnostic categories. Unlike the phases proposed by Robins and Guze, which function both as stages in a process of inquiry and as sources of evidence, Kendler’s validators are understood exclusively as types of studies capable of providing evidence relevant to diagnostic validity.

Kendler’s reconfiguration of the validation process also involved an expansion and further specification of the relevant study types, which he organized into eight validators grouped under three broader classes (1980, p. 700):

I. *Antecedent validators*
  A. Family studies
  B. Premorbid personality (including family type)
  C. Demographic factors
  D. Precipitating factors
II. *Concurrent validators*
III. *Predictive validators*
  A. Diagnostic consistency over time
  B. Other follow-up data
  C. Response to treatment

Within Kendler’s framework, the classification of validators as *antecedent*,* concurrent*, or *predictive* depends on whether they investigate periods prior to, at the time of, or subsequent to diagnosis. These three classes correspond to, and subsume, three of the phases originally proposed by Robins and Guze (1970), suggesting that Kendler’s validators were developed on the basis of the earlier framework. Accordingly, antecedent validators can be traced back to the family studies phase, concurrent validators to the laboratory studies phase, and predictive validators to the follow-up studies phase.

Although Kendler (1980) does not address this point explicitly, his conception of the validation process departs from that of Robins and Guze in that it appears to downplay the interconnected character of the different phases of validation and to focus primarily on one of them, namely, the testing of diagnostic categories against validator evidence. Even so, for Kendler the formation and selection of psychiatric constructs remain an important component of validation. Accordingly, in a later publication he characterizes *validation* as a process involving the generation of *nosological hypotheses* and their subsequent testing by means of *validator evidence*:

[A] scientific nosology would involve the generation of hypotheses about the… validity of competing diagnostic schemas. These hypotheses would be tested by the examination of the research data that addresses the given hypotheses to determine whether the individual hypothesis (e.g., diagnostic criteria A are more valid than diagnostic criteria B) is or is not supported by the available evidence. (1990, p. 970)

Notably, Kendler speaks here of the generation of *hypotheses* rather than of the formation of psychiatric categories or constructs. This reflects the fact that, for him, the object of validation is not limited to specific diagnostic constructs but also encompasses broader classificatory changes, such as treating a category as a subtype of a more inclusive one, splitting a category into multiple distinct categories, or integrating several categories into a higher-level diagnostic group.

Kendler’s (1980) motivations for developing this conception closely mirror those of Robins and Guze (1970). Like the Washington University group, Kendler (1990) understood his framework as a contribution to a broader program aimed at transforming psychiatric nosology into a genuinely scientific discipline. He likewise held that psychiatric classification had relied excessively on expert consensus rather than on rigorous empirical evidence, leaving it vulnerable to “parochial factors” and shifting “nosological fashions” (1990, p. 970).

Now, despite Kendler’s explicit rejection of the influence of “parochial factors” in psychiatric nosology, he ultimately acknowledges that, in some cases, validator evidence is insufficient to determine the validity of diagnostic categories. Kendler (1990, pp. 970–972) identifies several questions that arise during validation which cannot be answered solely on the basis of such evidence, including: *how to choose between alternative definitions of the same category when different validators are prioritized; which validators should be privileged when they support competing constructs; and how reliability should be weighed against validity in the assessment of diagnostic criteria*. In acknowledging these difficulties, Kendler concedes that his eight validators are not always sufficient to determine diagnostic validity and that, in such cases, nosologists must inevitably rely on *value judgments*, though he does not elaborate on the nature of these judgments nor on their epistemological implications. All in all, Kendler’s (1980, 1990) framework offers a more nuanced view than that of Robins and Guze (1970) regarding the extent to which validation can be grounded exclusively in empirical evidence.

Whether Kendler’s model should be regarded as a refinement of the framework originally proposed by Robins and Guze or as an entirely distinct method has received little attention in the literature. Nevertheless, most authors—including Kendler himself (1980, 1990, 2013)—have treated his proposal as a natural development and extension of the earlier approach (Regier et al., 2009; Schaffner, 2012; Solomon, 2022). More relevant for present purposes, is the fact that, since its initial publication in 1980, Kendler’s list of eight validators has gained considerable traction among DSM-oriented nosologists, being widely cited and even serving as a model for the guidelines currently employed by the committees and institutional bodies responsible for successive editions of the DSM (Kendler et al., 2009; APA DSM Team, 2021).

### Alternative models of validation

Alongside the frameworks proposed by Robins and Guze (1970) and later by Kendler (1980, 1990), Paul E. Meehl (1986, 1995) developed a markedly different approach to the validation of psychiatric categories, grounded in a theoretical orientation distinct from that of the Washington University group. Drawing on his previous work in psychometrics, Meehl imported the notion of *construct validity* into psychiatry. As originally defined by Cronbach and Meehl (1955), *construct validity* concerns the extent to which a test adequately measures an attribute that has not been operationally defined. Meehl’s approach, later termed “bootstrap taxometrics” (1995), consisted of a technically sophisticated set of statistical procedures designed to determine whether a psychiatric construct corresponds to a real categorical entity (a taxon) or to a dimensional continuum.

A central point of divergence between Meehl’s framework and that of the Washington University group concerns their respective stances toward the formation and selection of psychiatric constructs. Although they did not state it explicitly, Robins and Guze (1970) presupposed a particular structure for diagnostic categories during the phase of *clinical description.* Thus, in *Psychiatric Diagnosis*, Guze characterizes a diagnostic category as “a cluster of symptoms and/or signs with a more or less predictable course [that] may be associated with physical abnormality or may not” (Woodruff, Godwin, & Guze, 1974/1979, p. x). This model, together with paradigmatic cases such as dementia praecox and manic–depressive psychosis, was inherited from the disease model developed by the nineteenth-century German psychiatrist Emil Kraepelin, which emphasized differences in outcome as a criterion for distinguishing disorders. It is precisely because of the profound influence of Kraepelin’s work on the members of the Washington University group that they have often been described as “Neo-Kraepelians” (Klerman, 1978; Bashfield, 1984; Sadler, 2005).

By contrast, Meehl (1986, 1995) argued that validation should not begin with pre-established diagnostic categories whose validity is then tested against evidence. Influenced by the numerical taxonomy program of Sneath and Sokal (1973), which aimed to develop biological classifications free from theoretical presuppositions and grounded entirely in statistical analyses of trait similarity, Meehl maintained that validation should proceed from the bottom up. On his view, constructs are generated through the application of cluster algorithms to broad sets of relevant observations and the identification of patterns of covariation (*see also* Corning & Steffy, 1979).

A second point of contrast between Meehl’s framework and that of Robins and Guze concerns the tests employed to determine the validity of psychiatric constructs. As noted earlier, Robins and Guze (1970) identified three types of studies from which evidence relevant to diagnostic validity could be drawn—laboratory, follow-up, and family studies—a list that Kendler (1980) later expanded to include eight validators. Meehl, by contrast, did not commit himself to any predetermined set of study types. Instead, he proposed a set of statistical criteria that, he argued, could be applied to constructs from any domain, regardless of which study types were deemed relevant, in order to determine whether they correspond to real taxa (Meehl, 1995, p. 269).

Although Meehl’s taxometric approach was applied in several empirical contexts, most notably in his influential work on schizotaxia (Meehl, 1973, 1990), it failed to gain significant uptake among the bodies responsible for the development of *DSM-IV* and *DSM-5*. These committees largely continued to rely on the validation framework developed by Robins and Guze (1970) and later refined by Kendler (1980). This lack of uptake may be partly explained by limitations intrinsic to taxometric methods, such as the fact that evidence for the *taxonicity* of a construct, even when robust, remains largely silent with respect to its clinical utility, temporal stability, or underlying etiology (Watson, 2003; Trull & Durrett, 2005). However, it is also likely that broader theoretical commitments played a role: for a group trained within, and largely committed to, the Kraepelinian framework, adopting a blank-slate approach that bracketed prior nosological assumptions may have appeared as an excessively radical step, one they were neither inclined nor institutionally prepared to take.

Aside from Meehl’s work, relatively few alternative models of validation were proposed between the pubication of the *DSM-III* and the *DSM-5*. One notable example is the program advocated by Nancy Andreasen (1995, p. 161), which emphasized genetic studies, neuroimaging, and biological markers as complements to the project initiated by Robins and Guze (1970). However, this proposal remained largely programmatic and did not gain traction within the DSM revision process.

### The institutionalization of Kendler’s validators

As noted above, Kendler’s model of the validation process has been widely cited and, more importantly, incorporated into the official guidelines and institutional practices of the DSM, although not without modification. In the document “Guide to Submitting Proposals for Changes to DSM-5” (hereafter the *Guide*), the APA reproduces, with some adjustments, Kendler’s original list of validators (APA DSM Team, 2021, pp. 6–7):

1. Antecedent Validators
  Familial aggregation*
  Socio-demographic and cultural factors
  Environmental risk factors
  Prior psychiatric history

2) Concurrent Validators
  Cognitive, emotional, temperament, and personality correlates
  Biological markers*
  Patterns of comorbidity
  Degree or nature of the functional impairment*

3) Predictive Validators
  Diagnostic Stability*
  Course of illness*
  Response to treatment*

This list retains Kendler’s tripartite distinction between *antecedent*, *concurrent*, and *predictive* validators. While the antecedent and predictive categories remain largely unchanged, the category of concurrent validators is now subdivided into four specific types of studies. In addition, the document introduces a novel element absent from Kendler’s original framework: a subset of six validators, marked with an asterisk, that are designated as high priority. However, the criteria guiding this prioritization are not made explicit.

The *Guide* also retains and expands Kendler’s (1990) proposal that validation should combine evidence derived from validators with a set of additional considerations—which Kendler had described as *value judgments*. Accordingly, proposals for diagnostic changes are required not only to present relevant validator evidence but also to address further requirements, including “discussion of possible negative consequences”, “consideration of arguments against the proposed change”, and “evidence concerning reliability and clinical utility” (APA DSM Team, 2021, p. 12).

To understand how Kendler’s validators were incorporated into DSM-5 guidelines, it is useful to consider his own account in “A History of the DSM-5 Scientific Review Committee” (2013). Kendler explains that, after serving on both the *Task Force* and *Work Groups* for *DSM-III-R* (APA, 1987) and *DSM-IV* (APA, 1994), he was contacted in 2010 by the then president of the American Psychiatric Association, Carol Bernstein, and asked to chair a new committee charged with reviewing the scientific justification for proposed diagnostic changes in DSM-5. This committee was designed to function as an independent body alongside the APA Board of Trustees, which oversaw the overall revision process.^1^

In Kendler’s (2013) account, the DSM revision process had until then followed a model of “scientifically assisted consensus” that, although occasionally informed by literature reviews, relied primarily on the clinical experience of committee members for classificatory decisions. By contrast, the *DSM-5 Scientific Review Committee* (SRC) adopted what he describes as a “scientifically driven approach,” grounded in the document “Guidelines for Making Changes to DSM-V” (Kendler et al., 2009), of which he was one of the authors. This document—an early version of the *Guide* (APA DSM Team, 2021)—already contained the expanded list of eleven validators. Notably, however, Kendler (2013) does not indicate that alternative approaches to validation, such as Meehl’s, were considered in this context.

Following its publication, *DSM-5* (APA, 2013) adopted an iterative model of revision, under which proposals for diagnostic changes could be submitted whenever new validator evidence became available. Oversight of this ongoing process was assigned to the *DSM Steering Committee*, whose procedures and methodological approach were modeled on those of the *DSM-5 SRC.* In this light, Kendler’s prominent role in drafting the relevant guidelines and shaping the institutional practices of the *DSM-5 SRC* plausibly contributed to the adoption of his conception of validation—particularly his list of validators—within DSM guidelines and practices.

This historical reconstruction reveals a tension running through the articulation and development of the DSM validation method. On the one hand, the method emerged from, and continues to be oriented toward, the aim of grounding psychiatric classification in empirical evidence rather than in non-empirical considerations, an ideal first articulated by Robins and Guze (1970) and later reaffirmed by Kendler (1980, 1990) and the *DSM-5 SRC*. On the other hand, it was gradually recognized that empirical evidence alone was insufficient to address certain questions arising in the evaluation of diagnostic categories, a point already acknowledged by Kendler (1990) and subsequently endorsed in official DSM-5 guidelines (Kendler et al., 2009; APA DSM Team, 2021). This tension is further sharpened by the possibility that the development of the validation method itself was shaped, at least in part, by non-empirical factors, such as Kendler’s prominent institutional role in the formulation of the official guidelines and the Kraepelinian background commitments of the architects of the validation method.

What, then, should we make of this tension? Does it imply that the DSM validation method ultimately fails to deliver on the very aim that motivated its development—namely, the aspiration to ground psychiatric classification exclusively in empirical evidence? In the sections that follow, I address these questions by identifying the non-empirical factors that may have influenced the DSM validation method, specifying the roles they have played in its configuration, and assessing whether their incorporation should be regarded as epistemically pertinent or gratuitous.

## Non-empirical factor and decision points

Throughout the following sections, I will use the term “non-empirical factors” to refer to those elements that, in conjunction with validator evidence, guide the validation of psychiatric categories.^2^ I deliberately avoid the more common label “values” (e.g., Kendler, 1990; Sadler, 2005; Stegenga, 2011), because some of the factors examined here cannot be adequately captured by the standard notion of values in philosophy of science, which portrays them *as criteria of choice that influence*,* but do not determine*,* decisions*. On this traditional view, initiated by Thomas Kuhn (1977) and Ernan McMullin (1982), values are typically contrasted with the logical and statistical rules by means of which scientific hypotheses are assessed in light of the evidence, insofar as, unlike such rules, values are not only more open to interpretation but may also be weighed differently by different scientists. Within this perspective, values function as additional criteria that, in cases where the available evidence is insufficient, help guide decisions about whether to accept or reject a hypothesis under evaluation, or operate as *tie-breakers* in cases of underdetermination in which more than one hypothesis is compatible with the evidence (Elliott & McKaughan, 2014).

One important group of *non-empirical factor*s that does not fit this characterization of values, and that will be the focus of the following sections, consists of *metaphysical presuppositions*. Unlike values, metaphysical presuppositions do not function as evaluative criteria that scientific hypotheses or other epistemic products are expected to satisfy to a greater or lesser degree, but rather as substantive beliefs about the way the world is. As a result, although such metaphysical beliefs may be brought to bear in the assessment of scientific hypotheses and other scientific products, they are not employed in the same way as values, but instead operate as claims with which those hypotheses must be coherent. A similar distinction was drawn by McMullin, who argued that subsuming metaphysical (and theological) beliefs under the general category of values risks obscuring their distinctive contribution to scientific reasoning, namely, their role as considerations taken to be “truth-bearing reasons” for or against particular scientific hypotheses (1982, p. 703). McMullin also noted, however, that this way of deploying metaphysical and theological beliefs in scientific reasoning would be regarded as unacceptable by contemporary standards.

To identify the stages of the validation process at which non-empirical factors come into play, I will draw on the notion of *decision point* as developed in the literature on science and values. *Decision points* are moments in a process of inquiry at which epistemic agents must choose between multiple reasonable options, none of which is uniquely determined by the available evidence. Matthew Brown (2020), who has recently drawn attention to the importance of decision points in scientific practices, characterizes them as *unforced decisions* and *contingencies* (pp. 57–61): because they are not fixed by empirical or other epistemic factors, they could have been resolved in different, equally reasonable ways, each leading inquiry along a different trajectory. Moreover, Brown argues that there may be cases in which epistemic agents face a decision point even if they are not aware of it at the time, simply because more than one reasonable way of proceeding is available with respect to a particular aspect of an ongoing investigation. In such cases, it may appear that researchers have merely followed the natural course of inquiry, when in fact they have, by omission or by failing to consider the available alternatives with due care, effectively made a decision. Accordingly, what matters for identifying a decision point, on Brown’s view, is simply that more than one path could have been taken and that none is unambiguously determined by the available empirical evidence.^3^

For Brown (2020, pp. 63–65), the importance of *decision points* lies in the fact that they mark moments in the process of scientific inquiry at which the use of *value judgments* becomes necessary. When an agent is confronted with a choice for which the available evidence does not decisively favor one option over the others, the agent must weigh the implications of the available alternatives and thereby make a value judgment. Given that scientific inquiry routinely involves decision points of this kind, value judgments play a fundamental role in enabling inquiry to proceed. This, however, does not imply that all judgments made at decision points are thereby appropriate: the existence of a decision point entails only that a value judgment is required, not that it is epistemically or normatively warranted. Accordingly, applying the concept of *decision point* effectively requires distinguishing between two different questions: first, the question of the *epistemic pertinence* of incorporating value judgments into the process of inquiry, for which the notion of decision point is crucial; and second, the question of the *epistemic warrant and fruitfulness* of the particular value judgments that are introduced at such points.^4^

The notion of decision point also enables Brown (2020) to clarify the nature of value judgments and the role values play within them. On his account, value judgments are not mere expressions of researchers’ subjective preferences, desires, or emotions—a view widely held in philosophy of science and described by Brown as non-cognitivism (Brown, 2020, pp. 90–92). Instead, he thinks, they are judgments about which course of action to take when an epistemic agent is faced with multiple reasonable alternatives. In making such judgments, values function as criteria of choice that have certain influence over decisions, and in cases where different values support different options, value judgments may require weighing competing values against one another.^5^

The concept of *decision point* appears to underlie several accounts of the role that value judgments, and other non-empirical factors, play in scientific practice, even when it is not explicitly invoked. This is true both of approaches that, assuming some version of the *Value-Free Ideal*, regard such influences as threats to epistemic integrity (e.g., Lacey, 1999), and of those that take them to be compatible with the objectivity of scientific methods and practices (e.g., Longino, 1990; Douglas, 2009). Moreover, within the philosophy of biomedical science and the philosophy of medicine, a number of accounts of the role that non-empirical factors play in scientific research likewise presuppose some version of the notion of decision point (e.g., Sadler, 2005; Stegenga, 2011).

In the following sections, I will use the notion of a decision point—understood in a sense closely aligned with Brown’s characterization—together with the account of non-empirical factors developed here, to identify several factors that play an important role in the DSM validation method and to clarify the functions they serve within it. This analysis will be used to argue that the DSM validation method is not epistemically self-sufficient, in the sense of possessing all the resources required to address every form of indeterminacy it may encounter, but instead depends on the incorporation of non-empirical factors to resolve some of these situations. This, in turn, will allow me to argue for the *epistemic pertinence* of incorporating these two types of non-epistemic factors at specific decision points within the validation process. However, care will be taken to emphasize that an analysis in terms of decision points leaves open, and treats as a case-by-case matter, the question of whether particular decision points at which non-empirical factors were incorporated resulted in decisions that were *epistemically well-grounded* and *fruitful.*

## The role of value judgments in the validation process

In its current form, the DSM validation method, when applied to particular diagnostic categories or groups of categories, gives rise to *decision points*. Some of these had already been identified—albeit under a different terminology—by Kendler (1990), who noted that certain “questions” concerning the validation of specific categories cannot be resolved solely on the basis of validator evidence, but instead require recourse to value judgments. As mentioned earlier, these include *how to choose between alternative definitions of the same category when different validators are prioritized*,* which validators should be given precedence when competing constructs are supported by different sets of evidence*,* and how reliability and validity should be weighted in assessing diagnostic criteria.* The way Kendler describes both the emergence and resolution of these questions closely aligns with Brown’s characterization of decision points, insofar as they arise under conditions of evidential underdetermination and require judgment beyond what empirical evidence alone can determine.

This interpretation has been further reinforced by a recent analysis by Miriam Solomon and Kendler (2021). Although they do not employ the term “decision point”, their discussion highlights several stages of the validation process at which value judgments are required. In particular, they argue that the DSM validation method faces two central *problems* concerning the aggregation of psychiatric validator evidence: *(1) the absence of a principled basis for privileging some validators over others when they support competing constructs or nosological hypotheses*,* and (2) the apparent arbitrariness involved in setting evidential thresholds for determining when a construct should count as sufficiently validated.*

According to Solomon and Kendler (2021), the first of these problems stems from the fact that the DSM validation method operates as a *multimodal evidence-aggregation procedure* that integrates highly heterogeneous sources of evidence. Evidence-aggregation methods combine findings from different types of studies to assess whether a given hypothesis is supported by the total body of evidence (Stegenga, 2011; Greco et al., 2013; Holman, 2019). Unlike *meta-analyses* or *systematic reviews*, which typically focus on a single study type or a narrow range of designs, the DSM validation process incorporates evidence from eleven distinct validators, each encompassing multiple subtypes. Given this diversity, Solomon and Kendler argue, it is not only possible but to be expected that different studies will support competing constructs of the same diagnostic category or lend conflicting support to alternative nosological hypotheses (2021, p. 9).

To illustrate this problem, it is useful to consider a case discussed by Kendler (1990) involving two competing proposals for subdividing schizophrenia into subtypes. As Kendler explains, the subtypes proposed by Tsuang and Winokur (1974) performed better in terms of *reliability* and *follow-up* assessments, whereas those included in the 9th Revision of the *International Classification of Diseases* (WHO, 1978) exhibited greater *long-term diagnostic stability* and stronger *familial aggregation*. Because each proposal is supported by different validators, adjudicating between them requires determining which validators should be prioritized. According to Solomon and Kendler (2021), such cases cannot be resolved on the basis of empirical evidence alone, but instead depend on practical considerations and value judgments. In this way, the problem of conflicting validators closely fits the characterization of decision points developed above.

The second problem identified by Solomon and Kendler (2021) concerns how evidential thresholds should be set in order to validate a given construct or nosological hypothesis. Regardless of whether the available validator evidence is consistent or divided among competing alternatives, evaluation committees must ultimately determine whether the total body of evidence is sufficient to warrant a classificatory change. As with the problem of conflicting validators, Solomon and Kendler argue that the determination of evidential thresholds cannot be settled solely by empirical considerations. Rather, it involves assessing how much evidence should count as sufficient for validation in light of broader practical and evaluative concerns. In this sense, fixing evidential thresholds can also be understood as giving rise to a decision point in the sense characterized above.

That these two problems constitute decision points in the sense developed here is further reinforced by the conclusions Solomon and Kendler (2021) reach after examining a range of strategies for addressing them. Among the strategies they consider are Kendler’s (1980) informal aggregation procedure, which appeals to “the bulk of the evidence” to adjudicate between conflicting validators, and the prioritization strategy adopted in the DSM-5 guidelines, which assigns higher evidential status to a subset of validators (Kendler et al., 2009; APA DSM Team, 2021). Solomon and Kendler argue that both strategies remain problematic, insofar as they lack a clear methodological or epistemological justification and leave room for disagreement and arbitrariness.

A third strategy they discuss—particularly relevant for present purposes—consists in explicitly acknowledging the role of values in resolving conflicts among validators and in setting evidential thresholds. However, Solomon and Kendler do not specify how such values should be selected or how agreement about them might be achieved. In connection with this, Solomon (2022, p. 12) has recently argued that incorporating broader criteria, including values and considerations of utility, does little to resolve these difficulties and may even exacerbate them, given the absence of shared rules for selecting the relevant values. The fact that all available strategies are found to be unsatisfactory further underscores that the two problems of validator aggregation constitute genuine decision points: they are not resolved by empirical evidence alone, nor is there a principled way of settling them.

On this basis, Solomon and Kendler (2021) conclude that the problems of conflicting validators and of setting evidential thresholds place psychiatric validation in a situation analogous to the underdetermination of theory by evidence. Importantly, this diagnosis does not lead them to reject the DSM validation method. Rather, they maintain that, although imperfect, the aggregation of validator evidence remains functional, and they call for the development of more rigorous and reproducible procedures for managing the value judgments involved at these decision points (Solomon & Kendler, 2021, p. 12). At the same time, while their analysis significantly clarifies how value judgments enter the validation process, it does not exhaust the range of *non-empirical factors* or the *decision points* at which they may operate. In the next section, I therefore turn to a different class of non-empirical factors that can shape the decisions of nosologists and evaluation committees in the assessment of psychiatric categories.

## The roles of metaphysical presuppositions in the validation process

A second class of non-empirical factors that has significantly influenced the DSM validation method consists of *metaphysical presuppositions*. Unlike *value judgments*, which are required to resolve decision points arising in the validation of particular categories, metaphysical presuppositions function as *background principles* that shape the fundamental structure of the validation method and thereby indirectly influence the validation of specific diagnostic categories. The adoption and continued commitment to such principles can themselves be understood as outcomes of decision points.

The metaphysical presuppositions underlying the validation of psychiatric categories were not adopted as isolated beliefs, but as components of more *general models* of mental disorders. In psychiatric nosology, a *general model* can be understood as a set of claims and assumptions that offers a coherent picture of the fundamental features of mental disorders (Blashfield, 1984; Sadler, 2005). Such models incorporate theoretical and empirical components, but also include metaphysical claims concerning the causal structure of mental disorders and the boundaries separating disorders from one another and from states of mental health.

In the development of *DSM-III*, its subsequent editions, and their corresponding validation method, three general models were variously considered and adopted as background assumptions: the *medical model*, the *categorical model*, and the *dimensional model*. These conceptions, together with their associated metaphysical commitments, have shaped core features of DSM categories and the processes through which they are validated. Their incorporation into the background assumptions of the DSM validation method can therefore be understood as the result of a series of *decision points*, at which alternative ways of conceptualizing mental disorders were available, none of which could be selected on the basis of empirical evidence alone.

The *medical model* refers to a family of assumptions concerning the conditions something must satisfy to count as a disease or nosological entity. As John Sadler (2005, p. 89) notes, its core commitments include *naturalism*, *reductionism*, and *essentialism*, understood respectively as the view that diseases reflect natural disturbances, that these can be explained in terms of bio-psychological dysfunctions, and that disease entities possess an invariant underlying structure. These assumptions were explicitly endorsed by Robins and Guze (1970) and other members of the Washington University group, who argued that psychiatry should align itself with the rest of medicine by identifying discrete clinical entities grounded in biological causes (Woodruff et al., 1974).

The adoption of the medical model can plausibly be reconstructed as the resolution of a *decision point* in the development of the DSM and its validation method. In this case, however, the choice did not result from an explicit deliberation between equally developed general models. Rather, Robins and Guze (1970) appear to have adopted the medical model without seriously considering alternatives, either because these were not fully recognized at the time or because they were regarded as inadequate. The principal alternative available was not another substantive medical model, but Meehl’s a-theoretical approach, which sought to dispense with general models altogether and to ground psychiatric constructs in neutral statistical criteria (Meehl, 1986, 1995).

In this regard, some authors have suggested that Robins and Guze were likely familiar with Cronbach and Meehl’s (1955) work on *construct validity*, even though they did not cite it directly, and that this work probably influenced their conception of *diagnostic validity*, or at least their adoption of the language of validity and validation (Schaffer, 2012; Zautra, 2025). Thus, if we adopt the conception of decision points outlined above, the possibility that Robins and Guze were aware of an reasonable alternative approach to the medical model suffices to meet the minimal conditions for construing this situation as a decision point—namely, that decision-makers had more than one reasonable option available for how to proceed at a given juncture. In the case of the choice of the medical model over Meehl’s a-theoretical approach, it is likely that Robins and Guze’s judgment was influenced both by considerations concerning the costs—in time and resources—associated with the kind of fresh start proposed by Meehl, and by the continuity that the medical model afforded with respect to the Kraepelinian tradition, which likewise presupposed a broadly medical framework for understanding mental disorders.

One important consequence of adopting the *medical model* was its impact on the kinds of evidence regarded as appropriate for validation. As Sadler (2005, p. 87) observes, Robins and Guze’s (1970) commitment to this model led them to privilege *laboratory studies* as especially promising sources of validator evidence. Their description of this phase of validation reflects the assumption that mental disorders are syndromes caused by underlying biological mechanisms, and they expressed the expectation that advances in laboratory techniques would eventually yield more homogeneous, reliable, and valid diagnostic groupings, despite the lack of consistent laboratory findings at the time (Robins & Guze, 1970, pp. 983–984).

In this respect, the medical model, and the metaphysical presuppositions embedded in it, appear to have played a role analogous to that which Catherine Kendig attributes to metaphysical presuppositions in the evaluation of scientific classifications (Kendig & Grey, 2021; Kendig, 2022). On her account, such presuppositions help determine which entities, processes, or mechanisms are treated as salient when assessing alternative classificatory schemes. Similarly, Robins and Guze’s (1970) endorsement of the medical model shaped what counted as relevant validator evidence by privileging laboratory studies aimed at identifying underlying biological mechanisms.

A further aspect of the DSM validation method whose configuration involved a choice between general models of mental disorder concerned *how the boundaries of mental disorders were represented* in the construction of diagnostic constructs. Here, the relevant alternatives were the categorical and dimensional models. The *categorical model* characterizes mental disorders as discrete classes with well-defined boundaries, clearly separated from one another and from states of normality (Kendell & Jablensky, 2003; Sadler, 2005). By contrast, the *dimensional model* represents psychopathology in terms of quantitative dimensions that vary continuously across individuals, such as symptom severity or degrees of functional impairment (van Praag, 1987; Hyman, 2010). In short, whereas the categorical model draws qualitative distinctions, the dimensional model construes these differences as matters of degree.

The *categorical model* was ultimately privileged in guiding the construction and selection of the diagnostic constructs to be validated. This preference is evident in Robins and Guze’s (1970) expectation that laboratory studies would eventually identify etiological factors *specific* to individual diagnostic categories, thereby reinforcing the assumption that psychiatric disorders are discrete disease entities. The adoption of the categorical model had important downstream implications for the DSM validation method: it oriented the selection of constructs toward those amenable to categorical articulation and shaped expectations about the results validation should deliver, namely, the identification of “zones of rarity” between categories, i.e., discontinuities in symptom distributions taken to mark natural boundaries between disorders and between disorder and health. As Robert Kendell and Assen Jablensky state it:

The weakness of the validity criteria of both Robins and Guze and Kendler was that those criteria implicitly assumed that psychiatric disorders are discrete entities and that the role of validity criteria is to determine whether a putative disorder… is a valid entity…. The possibility that disorders might merge into one another with no natural boundary in between called a “point of rarity,” but what is better regarded as a zone of rarity—was simply not considered. (Kendell & Jablensky, 2003, p. 5)

The choice between the categorical and the dimensional model can be adequately represented as a *decision point* in the sense articulated above. At the time when the DSM validation method was taking shape, there appear to have been no decisive empirical grounds for preferring one model over the other. Substantial bodies of evidence bearing on their respective adequacy (such as findings concerning zones of rarity, comorbidity, and continuity between disorder and normality) would only become available decades later. Moreover, this choice could not have been settled by appealing to background assumptions of the DSM validation method, since both models were, in principle, equally compatible with the *medical model*. This is reflected in general medicine, where disease entities are represented both categorically (e.g., pneumococcal pneumonia) and dimensionally (e.g., dyslipidemias and hypertension) (Hyman, 2010, p. 163).^6^ Precisely, because there were neither decisive empirical grounds nor clear epistemic criteria for adjudicating between these models, Steven Hyman (2010) characterizes this choice as a “contingent top-down decision,” closely paralleling the notion of decision point defended here. In this sense, selecting a framework for representing the boundaries of mental disorders required value judgments in which decision-makers weighed the respective costs and benefits of each model in light of practical, institutional, and social considerations.

It is not entirely clear which considerations guided Robins and Guze’s (1970) decision to privilege the categorical model. Nevertheless, the continued commitment to this model—despite the accumulation of evidence favoring dimensional approaches—reveals the influence of practical and institutional factors. This is evident in the deliberations of the *DSM-IV* and *DSM-5* work groups reviewing proposed changes to the classification of personality disorders, where concerns about institutional costs and anticipated resistance from clinicians played a central role in rejecting dimensional proposals (First, 2010, p. 470). Similarly, Kendell and Jablensky (2003) and Hyman (2010) note that the entrenched use of categorical diagnoses across research, clinical training, insurance systems, and regulatory frameworks created strong incentives to preserve existing categories. Tsou (2021) further argues that broader social and pragmatic considerations, including perceived clinical utility, were decisive in mantaining the categorical model.

Although the decision points associated with the choice of general models of mental disorder do not belong to the validation of particular diagnostic categories strictly speaking, they have nonetheless exerted a significant influence on the DSM validation method. By shaping its background assumptions, these decisions have affected the design of core elements of the validation process, including which types of constructs are eligible for validation and which forms of evidence count as validators. As a result, the adoption of specific general models has produced downstream effects that shape and constrain the conditions under which individual diagnostic categories are assessed, thereby indirectly influencing validation outcomes.

## Taking stock of decision points

From the analysis in terms of the notion of *decision point* developed here, it is possible to address the tension between the aim of grounding psychiatric classification solely in empirical evidence and the fact that non-empirical factors have played a significant role in the construction and application of the DSM validation method. By characterizing the incorporation of value judgments and metaphysical presuppositions as arising from decision points their inclusion can be understood as a necessary response to the circumstances under which the DSM validation method was designed, to the nature of the scientific products it evaluates, and to the kinds of evidence available for doing so.

Thus, in the case of the questions identified by Kendler (1990) and of the problems that Solomon and Kendler (2021) diagnose in the aggregation of psychiatric validators, insofar as these situations fit the notion of decision point articulated here, the incorporation of value judgments guided by practical considerations can be seen as a necessary condition for the validation process to proceed and reach a conclusion. Moreover, in one of these cases—the fixing of evidential thresholds for the validation of diagnostic constructs—the reliance on value judgments appears to correspond to what some philosophical accounts regard as a *legitimate role* for values in scientific reasoning. In this respect, Heather Douglas (2009) argues that value judgments can appropriately guide the setting of evidential standards through assessments of the risks associated with erroneous acceptance or rejection.

A similar assessment applies to the decisions to adopt the medical model over Meehl’s a-theoretical approach and the categorical model over the dimensional one, both of which can be characterized as outcomes of decision points in the sense developed above. In these cases, the choice between *general models*—or the decision to dispense with them altogether—was a necessary step in the initial development of the validation method, insofar as it provided a provisional framework for determining which types of studies would count as validators and for guiding the formation of diagnostic constructs, at a time when available evidence was limited and many of the study types now commonly employed did not yet exist.

That said, the continued preservation of the categorical model in the face of an apparently growing body of validator evidence against it illustrates a case in which non-empirical factors appear to have been used in a problematic way. One consequence of adopting the categorical model was the expectation that zones of rarity would be found between disorders and between disorder and mental health. However, evidence from multiple validators has failed to support this expectation: high levels of comorbidity and continuity with normal functioning instead suggest that many mental disorders are better conceptualized dimensionally (Kendell & Jablensky, 2003; Hyman, 2010; Tsou, 2021). If this assessment is correct, the continued preservation of the categorical model despite contrary evidence would amount to what has been described as an illegitimate use of value judgments, namely, allowing values to substitute for evidence in the evaluation of scientific hypotheses (Douglas, 2009). One might therefore argue that, given the availability of relevant validator evidence, this choice would no longer qualify as a decision point in the sense employed here. Nevertheless, whether the existing evidence is sufficient to warrant replacing the categorical model with a dimensional one remains an open question, a situation that could itself give rise to a new decision point (Frances, 1982, 1993; Zachar, 2014).

The fact that the DSM validation method requires value judgments to resolve specific questions should therefore not come as a surprise; rather, it serves to further support influential accounts of the role of epistemic and non-epistemic values in scientific reasoning, as developed by Kuhn (1962[2009], 1977) and McMullin (1982), and further elaborated by Douglas (2009) and Brown (2020). In closely related methodological contexts, Stegenga (2011, 2018) and Holman (2019) have similarly argued that meta-analysis, as a method for aggregating evidence, involves decision points that require value judgments, even though they diverge in their assessments of the implications of this fact for methodological legitimacy.

In this way, the analysis in terms of decision points allows a case to be made for the epistemic pertinence of incorporating value judgments and metaphysical presuppositions into the design and application of the DSM validation method, while leaving open the question of the warrant of how particular decision points are resolved. If this analysis is sound, an important implication follows for how the aim of the DSM validation method should be understood. The formulation of this aim by Robins and Guze (1970), and later by Kendler (1990), as grounding psychiatric classification in empirical evidence as opposed to non-empirical factors appears to have been unrealistic. A more adequate formulation is that *validation should be grounded primarily in empirical evidence*,* while incorporating non-empirical elements only when decision points arise that would otherwise prevent the process from advancing and reaching a conclusion*.

## Conclusion

The analysis developed here has aimed to show that the incorporation of non-empirical factors into the DSM validation method can, under certain conditions, constitute a necessary and pertinent component of the validation process. I argued that such conditions initially arose when the validation method was still at an early stage of development and neither well-established bodies of evidence nor clearly defined sources of evidence were available, making it necessary to adopt general conceptions—which involved metaphysical assumptions about the structure of mental disorders—in order to determine which methods of evidence generation were most promising. A second set of conditions arises when the available evidence is heterogeneous and supports competing constructs or hypotheses concerning the same category, or when it is unclear how much evidence is required to establish a diagnostic construct as valid.

Nevertheless, this does not mean that any decision concerning the design or application of the validation method that relies on non-empirical factors is thereby warranted. Whether such decisions are well-grounded must be assessed on a case-by-case basis. In some instances, one can identify what appears to be a legitimate use of value judgments, as in the setting of evidential standards for validating specific diagnostic categories; in others, non-empirical factors seem to function not as auxiliaries to empirical evidence but as considerations that substitute for, or even run counter to, it. A rigorous evaluation of such cases, which lies beyond the scope of this paper, would require the development of a substantive normative framework specifying the conditions under which non-empirically informed decisions in psychiatric validation can be regarded as warranted. Moreover, the analysis of value judgments and metaphysical presuppositions offered here is not intended to be exhaustive, and other types of non-empirical factors may also influence the DSM validation method in ways not considered in this paper.

## Footnotes

1 Several entities are involved in the DSM revision process, each of which fulfills distinct roles. For every new edition of the DSM, a *Task Force* oversees the development of the entire edition, while several *Work Groups* are responsible for the sections corresponding to specific clusters of diagnostic categories (APA, 2022). On the other hand, the *Board of Trustees* is the APA’s governing body that holds the final authority in approving a new edition of the DSM (APA, 2022). For DSM-5, however, a special body, *the DSM Scientific Review Committee*, was created to evaluate the scientific evidence supporting proposed changes; this committee was later replaced by the *DSM Steering Committee* (APA, 2022).

2 In adopting this terminological choice, I follow a line of thought similar to that developed by Hannah Hilligardt (2022), who argues that an exclusive focus on values has obscured the ways in which broader influences shape scientific practices, and that values constitute only one subtype of non-epistemic factors (Hilligardt, 2022, p. 58). In addition to values, Hilligardt explicitly identifies other relevant influences that are not themselves values, such as social perspectives, opinions, and interests. Nevertheless, I adopt the label “non-empirical factors” rather than Hilligardt’s preferred terminology, since the contrast I seek to draw is with the empirical evidence provided by validators, rather than between internal and external factors—as the term “contextual” may suggest—or between epistemic and non-epistemic values.

3 Among the decision points that, according to Brown, may arise over the course of a process of inquiry are the framing of the problem to be investigated, the concepts and categories employed in the analysis, the hypotheses to be adopted, the methods used for data collection and the ways in which data are characterized, and the decision on how strongly the available evidence supports or challenges a given hypothesis (Brown, 2020, pp. 58–59).

4 From Brown’s (2020, p.162) perspective, addressing this second question requires examining whether the available options were assessed in a balanced manner and whether the resulting judgment contributed to the success of the inquiry.

5 Brown’s (2020) conception of value judgments is deeply influenced by John Dewey’s (1915, 1939) view of valuation as an integral component of inquiry, as well as by the work of philosophers who have drawn on Deweyan insights to develop contemporary alternatives to non-cognitivist accounts of values, such as Elizabeth Anderson (2004) and Mark Johnson (2014).

6 Meehl explicitly adopted a neutral stance with respect to the categorical–dimensional distinction, holding instead that whether the latent structure of a construct is categorical or dimensional must be determined on a case-by-case basis (Meehl, 1995, p. 266).
